# GrlJ, a *Dictyostelium *GABA_B_-like receptor with roles in post-aggregation development

**DOI:** 10.1186/1471-213X-7-44

**Published:** 2007-05-14

**Authors:** Yogikala Prabhu, Rolf Müller, Christophe Anjard, Angelika A Noegel

**Affiliations:** 1 Institute of Biochemistry I, Centre for Biochemistry, Medical Faculty, University of Cologne, Joseph-Stelzmann-Str. 52, D-50931 Köln, Germany; 2 Centre for Molecular Medicine Cologne, University of Cologne, Joseph-Stelzmann-Str. 52, 50931 Köln, Germany; 3 Center for Molecular Genetics, Division of Biological Sciences, University of California San Diego, La Jolla, CA 92093-0368, USA; 4 Cell Biology and Metabolism Branch, National Institute of Child Health and Human Development, National Institutes of Health, Bethesda, MD 20892, USA

## Abstract

**Background:**

The G-protein-coupled receptor (GPCR) family represents the largest and most important group of targets for chemotherapeutics. They are extremely versatile receptors that transduce signals as diverse as biogenic amines, purins, odorants, ions and pheromones from the extracellular compartment to the interior via biochemical processes involving GTP-binding proteins. Until recently, the cyclic AMP receptors (cARs) were the only known G protein coupled receptors in *Dictyostelium discoideum*. The completed genome sequence revealed the presence of several families of GPCRs in *Dictyostelium*, among them members of the family 3 of GPCRs, the GABA_B_/glutamate like receptor family, which in higher eukaryotes is involved in neuronal signaling.

**Results:**

*D. discoideum *has seventeen Family 3 members of GPCRs, denoted GrlA through GrlR. Their transcripts are detected throughout development with increased levels during early and late development. We have examined here GrlJ. GFP-tagged GrlJ localises to the plasmamembrane and to internal membranes. Inactivation of the grlJ gene leads to precocious development, and the mutant completes development ~6 hours earlier. Alterations were also noted at the slug stage and in spore formation. *grlJ*^- ^slugs were longer and broke apart several times on their way to culmination forming smaller but proportionate fruiting bodies. Spores from *grlJ*^- ^fruiting bodies were malformed and less viable, although the spore differentiation factors were synthesized and sensed normally. Expression of a GFP-tagged full length GrlJ rescued the phenotype.

**Conclusion:**

Our data suggest that GrlJ acts at several stages of *Dictyostelium *development and that it is a negative regulator in *Dictyostelium *development.

## Background

The seven-transmembrane spanning G protein-coupled receptors (GPCRs) represent a major group of cell-surface detectors and constitute 3.5 % of the genome in vertebrates [1]. They play a key role in the physiology of multicellular organisms as they transduce a broad variety of extracellular signals into the cell. Of the 1000 genes thought to encode GPCRs in humans, about 300–400 mediate effects by endogeneous ligands, with the remainder being sensory receptors. GPCRs can be grouped into six families based on the sequence similarity of their common heptahelical domain (7TM). Four of the families are present in *Dictyostelium discoideum*.

*D. discoideum*, a soil-living amoeba feeds on bacteria in decaying vegetation and reproduces by binary fission. Growing amoebae chemotax toward folic acid and other nutrients, whereas starved cells aggregate by chemotaxis toward cAMP. cAMP is sensed by cAMP-receptors, which form an unusual family of GPCRs [2]. Signalling via cARs is mediated by Gα2, one of the 14 Gα proteins of *D. discoideum*, however, many cAMP responses are also independent of G proteins [3]. Several other signaling molecules such as cell density factors, folate and related pterins, LPA (lysophosphatidic acid), PSF (prestarvation factor), CF (conditioned media factor), small metabolites and numerous small peptides are known to be acting via GPCRs as well [4,5]. Their receptors have not been identified so far.

The family 3 (or C) of GPCRs contains receptors for a wide range of signals: for the main neurotransmitters glutamate and γ-aminobutyric acid, for Ca^2+^, for sweet and amino acid taste compounds, for pheromones and for odorants. Accordingly they fall into six groups, the metabotropic glutamate receptors, the Ca^2+^-sensing receptors, the γ-aminobutyric acid_B _receptors, the pheromone receptors, the sweet and amino acid receptors and the orphan family 3 receptors. They possess a seven transmembrane domain (HD or 7TM) responsible for G-protein activation, and a large extracellular domain responsible for ligand binding. This domain is similar to bacterial periplasmic proteins that are involved in the transport of small molecules, and is called Venus Flytrap module (VFTM). The metabotropic GABA_B_/glutamate like receptor family was considered animal-specific and was not found outside the metazoan branch until the *Dictyostelium *genome was sequenced [2,6,7]. Altogether, 17 genes encoding GABA_B _like receptors were identified based on the homology of the HD. They are named GrlA through GrlR (**G**ABA_B _or metabotropic **g**lutamate – **r**eceptors **l**ike) proteins (Dale Hereld, *Dictyostelium *Genomics, 2005).

In the present study we undertook an investigation of GrlJ, located on chromosome 2 [6]. GrlJ is expressed throughout growth and development with a strong increase in development. It has a crucial role at several stages of development leading to precocious development, the formation of longer slugs that break apart several times while migrating and to the production of spores that have an abnormal morphology and exhibit reduced viability.

## Results

### *Dictyostelium *family 3 of GPCRs

The *Dictyostelium *genome possesses 17 genes encoding receptors belonging to the Family 3 of GPCRs and all of them resemble GABA_B _or metabotropic glutamate like-receptors [2]. They are represented as Grl (metabotropic **G**ABA_B _or **g**lutamate – **r**eceptor **l**ike) proteins. Grl encoding genes are present on all chromosomes. Some of them such as GrlA, B and O are close together. Their molecular masses range from 78.000 for GrlL to 181.000 for GrlR. However most of them have masses below 100.000 and only GrlP, Q and R are significantly larger. The increase in mass is mainly due to an extended N-terminal region. Most of them have a signalpeptide and a BMP (basic membrane protein) domain in their amino terminal region, a domain which was first found in outer membrane proteins of bacteria, and which is similar to the VFTM that forms the ligand binding site in the N-termini of metabotropic GPCRs [8]. Exceptions are GrlE, N, P, Q and R (Table 1). The N-terminal region of GrlE has homology to the mouse and the *Dorsophila *glutamate receptor and was identified as a true GABA receptor in *D. discoideum *[9,10]. GrlP, Q and R have a region of homology with *Chlamydia *polymorphic membrane protein in their amino terminal sequences.

**Table 1:** The Grl protein family in *D. discoideum*.

**Grl**	**Chrom**	**Position-**	**Length AA**	**kDa**	**BMP**	**Signalpeptide**
**A**	2	765675..769136	798	89.6	+	+
**B**	2	762281..765560	755	84.1	+	+
**C**	3	5788981..5792462	800	89.5	+	+
**D**	4	5037360..5040807	791	87.7	+	+
**E**	6	173112..176678	816	89.8	-	+
**F**	3	5486880..5490291	770	86.4	+	+
**G**	2	1727422..1730803	772	86.3	+	+
**H**	3	5785001..5788349	764	84.8	+	+
**J**	2	1586617..1590412	783	87.5	+	+
**K**	1	2668008..2671080	704	78.3	+	-
**L**	3	4210326..4213465	708	78.0	+	+
**M**	4	4777729..4781050	749	82.6	+	+
**N**	4	1197650..1201654	891	100.6	-^1^	-
**O**	2	758954..762730	819	92.6	+	-
**P**	5	4985774..4992939	1407	155.8	-^2^	-
**Q**	5	758954..762730	1095	124.5	-^2^	-
**R**	5	553899..560973	1604	181	-^2^	-

^1^GrlN habors an additional predicted transmembrane domain in its N-terminal region.^2^GrlP, Q and R have long N-termini which have a region of homology with *Chlamydia *polymorphic membrane protein.

**Figure 1:**
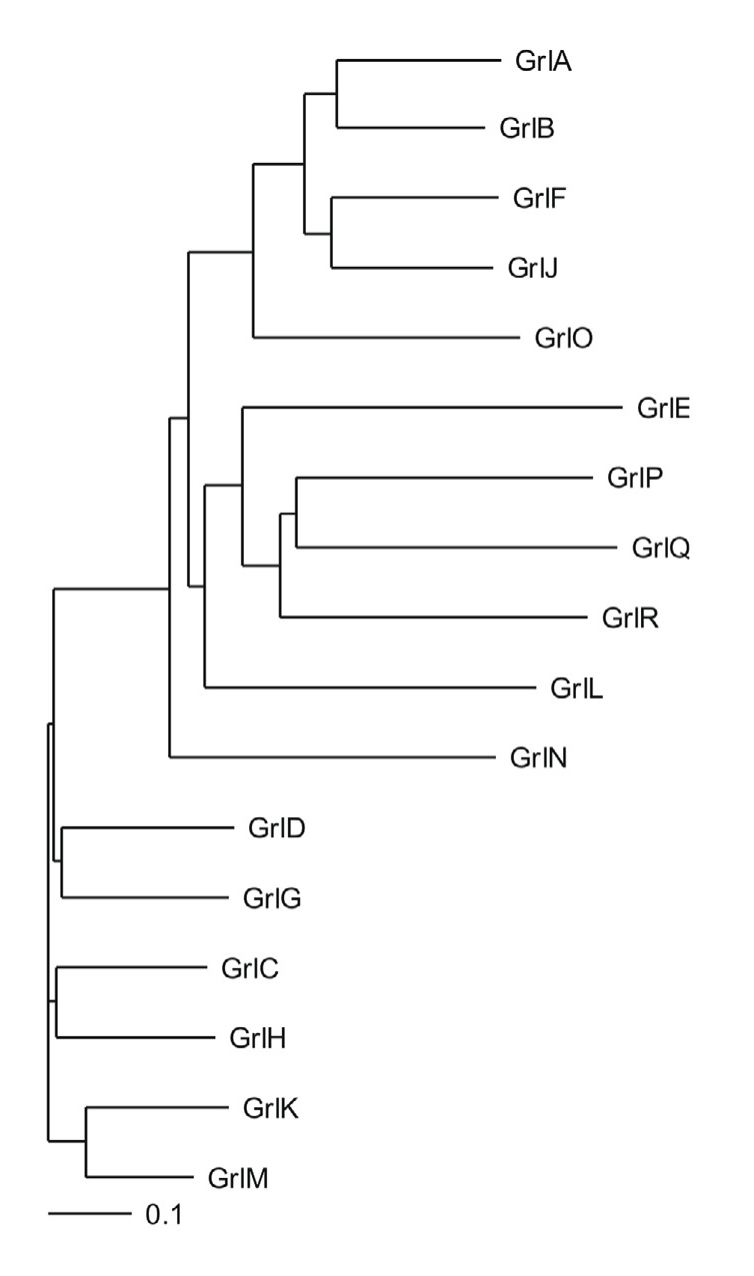
**Phylogenetic tree of the GABA_B _receptor-like proteins (Grl) in *D. discoideum***. The dendrogram was constructed with ClustalX using the sequence information of all Grls in the *Dictyostelium *database, the image was generated with the TreeView programme. Bar, 0.1, represents the phylogenetic distance (percentage of divergence divided by 100).

In a phylogenetic tree that was constructed using the full length sequences of all Grl receptors three major clusters were formed, consisting of GrlA, B, F, J in one group, GrlP, Q, R forming the second and GrlC, D, G, H, K and M forming the third and largest one. Four of the Grls (GrlO, GrlE, GrlN and GrlL) diverted out separately (Fig. 1). When analysing the transcript levels of all Grls throughout the developmental stages by semiquantitative RT-PCR analysis we detected the transcripts for all genes at all times. We found increased levels from early aggregation (GrlA, D, E, J, M, N, Q) or the tight aggregation stage onward (GrlB, C, F, G, H, K, L, O, P, R), when the contact site A is expressed. The levels stayed high during late development and transcripts were clearly present at t24 when fruiting body formation is close to completion. GrlA transcripts were still quite abundant at this stage (Table 2). Additionally we quantified the mRNA amounts for GrlJ using the actin mRNA for comparison. We found that the GrlJ message is of very low abundance throughout development ranging from 2.8 to 11.1 pg, whereas for actin we obtained amounts between 382 and 605 pg in these samples (Table 3).

**Table 2:** Expression of *grl *genes during growth and development.

**Grl**	**0 hrs**	**4 hrs**	**8 hrs**	**12 hrs**	**14 hrs**	**18 hrs**	**24 hrs**
**A**	+	+	++	+++	+++	+++	++
**B**	+/-	+	+	+++	++	++	+
**C**	+	+	+	+++	++	++	+
**D**	+	+	++	+++	+++	++	+
**E**	+	+	++	+	++	+	+
**F**	+	+	+	+++	+	+	+
**G**	+	+	+	++	+	+	+
**H**	+	+	+	++	++	++	+
**J**	+	+	++	++	++	++	+
**K**	+/-	+	+	++	+	+	+
**L**	+	+	+	+++	++	++	+
**M**	+/-	+	++	+++	+++	+++	+
**N**	+	+	++	++	++	++	+
**O**	+	+	+	++	++	++	+
**P**	+/-	+/-	+	+++	+	++	+
**Q**	+	+	++	+++	++	++	+
**R**	+/-	+	+	+++	++	++	+

A summary of results from RT-PCR is shown. Respective Grls are indicated in the left column and hours of development in the top row. Key: +++: strong band; ++: clear band; +: weak band; +/-: hardly detectable; -: no expression detected. The measurements were done in a qualitative RT-PCR as described in Methods.

**Table 3:** GrlJ mRNA quantification as determined by Real-Time RT-PCR (qRT-PCR)

**Time points (h)**	**GrlJ (pg)**	**GrlJ (relative levels)**^1^	**Actin control (pg)**
0 h	4.0	1	502
4 h	2.8	0.85	412
8 h	3.6	1.18	382
12 h	9.9	2.30	539
16 h	10.8	2.25	605
20 h	11.1	2.85	487

RNA was isolated from various developmental stages (given in hours). ^1^The relative levels of GrlJ during development were determined assuming that the level of the actin message is constant throughout development. The level of GrlJ at the 0 hour time point was arbitrarily set to 1.

GrlJ is a 783 amino acid containing protein possessing a typical signal sequence for secretion followed by a Bmp (Basic membrane protein) domain and a seven transmembrane domain. The fold of the Bmp domain is similar to the fold of the bacterial periplasmic binding proteins (PBP) which function in scavenging or sensing nutrients in the environment [11]. Sequences coding for PBP domains can occur in combination with diverse sequences coding for integral membrane domains. In 7 transmembrane receptors they act as the extracellular ligand binding site such as in the Glutamate/glycine gated channels, metabotropic glutamate/GABA_B_, Ca^2+^sensing, pheromone and atrial natriuretic peptide-receptors [12].

**Figure 2:**
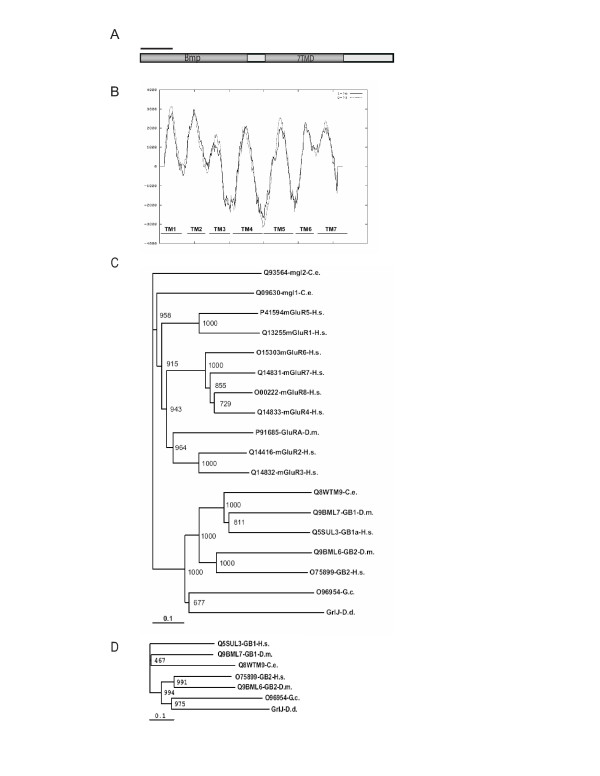
**(A) Schematic representation of GrlJ**. The conserved domain database [51] and SMART [52] was used to predict conserved domains for the protein sequence and drawn to scale. Bmp, basic membrane protein; 7TMD, seven transmmembrane domain. (**B) Hydropathy analysis of GrlJ**. The putative transmembrane regions (amino acid residues 375–638) were used to deduce the hydrophobicity of GrlJ using the TMpred programme [53]. Positive scores indicate a membrane spanning potential which predicted the presence of 7 transmembrane regions (TM1, TM2, TM3, TM4, TM5, TM6, TM7). (**C) Phylogenetic tree of *Dictyostelium *GrlJ and metabotropic GABA_B _and glutamate receptors from other eukaryotes**. A CLUSTALX alignment of the full length sequence of *Dictyostelium *GrlJ and selected GABA_B _and glutamate receptors from other organisms was applied to the TreeView program to obtain a phylogenetic tree. The scale bar indicates 10% divergence. The proposed names were used for the *Dictyostelium *receptors and UniProt identifiers for the receptors from other organisms. C.e.: *Caenorhabditis elegans*; H.s.: *Homo sapiens*, D.m.: *Drosophila melanogaster*, G.c: *Geodia cydonium*. (**D) Phylogenetic tree of the transmembrane regions of *Dictyostelium *GrlJ and metabotropic GABA_B _receptors from other eukaryotes**. Analysis of the transmembrane regions of the respective genes was carried out as described above applying the CLUSTALX and TreeView programme. Scale bar, 10% divergence.

While the full length sequence of GrlJ appears closer to GABA_B _receptors than other characterised members of the family 3, a detailed analysis revealed that the similarity was pertaining specifically to the transmembrane regions (Fig. 2A–C). GrlJ clustered more closely to the GABA_B_R2 subtypes in a phylogentic analysis that was performed with only the transmembrane domains (Fig. 2D). 13 members of the *Dictyostelium *Family 3 GPCRs share this domain organisation with GrlJ. GrlN, P, Q and R are different (Table 1).

### Subcellular localisation of GrlJ

**Figure 3:**
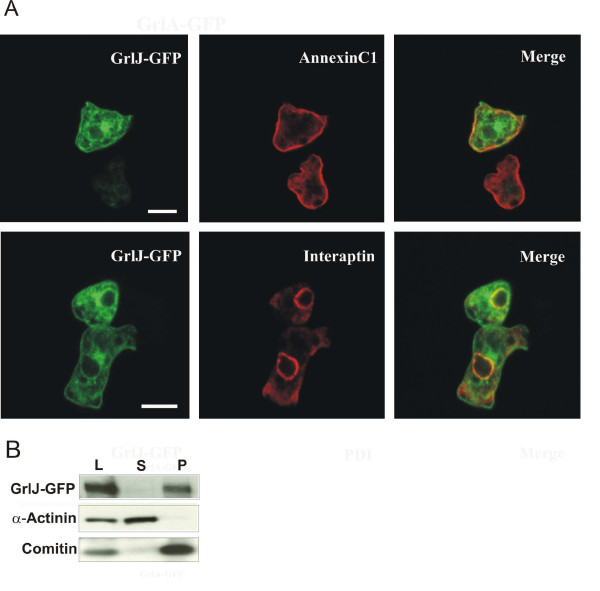
**Cellular localisation of GrlJ**. **(A) A GFP-tagged GrlJ fusion protein localises to the plasma membane and intracellular membranes**. GrlJ sequences were fused to GFP sequences in such a way that GFP was present at the C-terminus of GrlJ. Shown are *grlJ*^- ^cells expressing GrlJ-GFP. The cells were fixed with cold methanol and stained for annexinC1 using mAb 185-338-2 [13] or interaptin using mAb 260-60-10 [15]. Detection was with Cy3-labeled secondary antibody. Confocal images are shown. Bar, 10 μm. **(B) Distribution of GrlJ-GFP in cell fractionation studies**. *grlJ*^- ^cells were lysed by sonification and the post nuclear supernatant separated into supernatant and pellet by centrifugation at 100.000 g. The proteins were separated by SDS-PAGE (10% acrylamide), the resulting western blot was probed with GFP-specific antibody K3-184-2. α-Actinin, a cytosolic protein, and Comitin, a membrane associated protein, were used for control [14]. L, whole cell lysate, S, 100.000 g supernatant, P, 100.000 g pellet.

The subcellular localisation of GrlJ was studied by expressing a full-length GrlJ-GFP fusion protein. The expression of the fusion protein was under control of the actin15 promoter which is a strong promoter and will lead to an overexpression. GrlJ-GFP was present at the plasma membrane, the majority of the protein was however found on internal structures. At the plasma membrane GrlJ-GFP overlapped to a good extent with Annexin C1, a protein present at the plasma membrane and at internal membranes [13,14]. In the cell there was partial colocalisation with interaptin, a protein found at the nuclear envelope and the ER [15] (Fig. 3A). We also monitored for colocalisation of GrlJ-GFP with antibodies against VatA, which is present on endosomal membranes and the contractile vacuole of *D. discoideum *(A subunit of vacuolar ATPase) [16], lysosomal antigen (mannose-6-sulfate containing carbohydrate epitope present in lysosomal enzymes) [17]], and Vacuolin [18], a protein on post lysosomal vacuoles. However, we did not note colocalisation with any of these proteins (data not shown). The presence of GrlJ-GFP on ER- and Golgi-membranes may may reflect the transport pathway of the protein towards the plasma membrane. An analysis of the GrlJ-GFP localisation during development (6 h and 16 h) did not indicate changes in its subcellular distribution (data not shown). Western blot analysis of subcellular fractions of GrlJ-GFP expressing cells supported a membrane association as GrlJ-GFP was exclusively present in the 100.000 g pellet like the Golgi component comitin (Fig. 3B).

### GrlJ gene disruption and mutant behaviour

**Figure 4:**
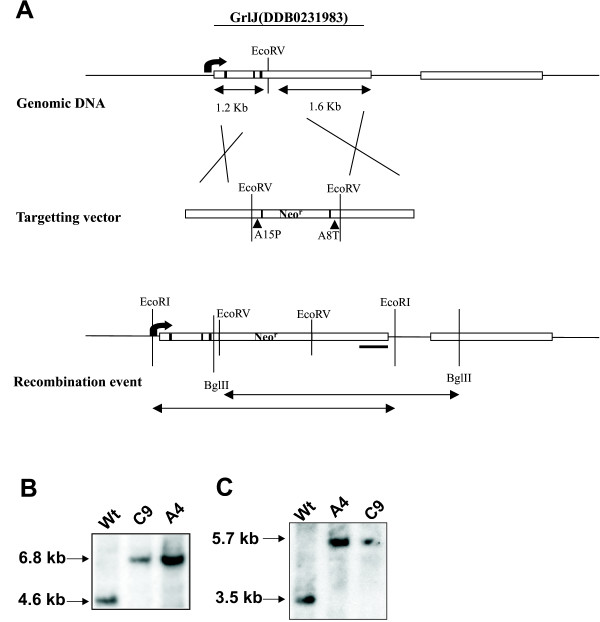
**Generation of *grlJ*^- ^strains. (A) Targeting vector and recombination strategy**. *Gene disruption vector for GrlJ: *The neomycin resistance cassette (2.2 kb) was obtained from pDNeo2 by EcoRV digestion and cloned into the EcoRV site of the GrlJ gene that was present in the pGEMTeasy vector and the DNA fragment was used for transfection of A×2 cells. (**B, C) Confirmation of the recombination event**. The recombination event of *grlJ*^- ^was analysed by Southern blotting. Transformants were selected with 4 μg/ml G418 for Neomycin resistance. Single colonies were obtained by spreader dilution of the whole pool of transformants onto SM agar plates overlaid with *Klebsiella areogenes*. Single transformants were then grown with the respective selection medium in a 96-well plate and eventually transferred to a 24-well plate and a 6-well plate. The amoebae were spread on *K. aerogenes *again and used to isolate genomic DNA for Southern blot analysis. The DNA was digested with either BglII (**B**) that has recognition sites in the GrlJ gene near the N terminus and 3' of the end of the gene or with EcoRI (**C**) that has recognition sites outside of the GrlJ gene. Separation of the DNA was in agarose gels (0.7 % agarose). The resulting blot was probed with a C-terminal probe indicated by a bar. WT, A×2; C9 and A4, two independent transformants.

**Figure 5:**
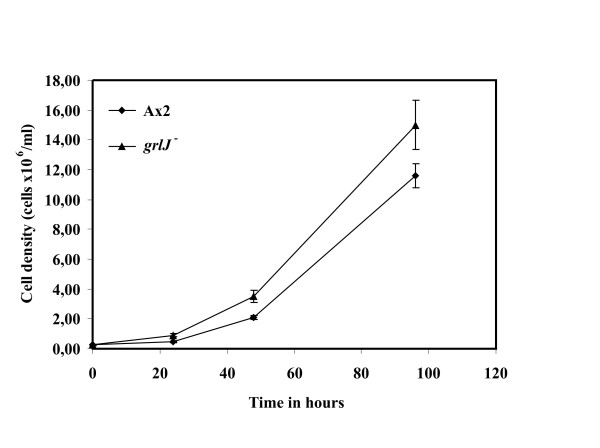
**Mutant analysis. Growth in axenic medium**. Log phase wild type and mutant cells were inoculated in equal volume of medium at a density of 2 × 10^5 ^cells/ml and grown at 21°C with shaking at 160 rpm. Cell numbers were determined at different time points. At the time point 100 hours both strains had reached the stationary phase.

To gain insight into the *in vivo *functions of GrlJ, mutants carrying a disruption of the *grlJ *gene were generated (Fig. 4). Growth of *grlJ*^- ^was not affected and the mutant reached slightly higher cell densities when grown in suspension culture in axenic medium as compared to the wild type A×2 (Fig. 5). On a lawn of *Klebsiella aerogenes *growth was comparable. Cytokinesis and cell size were unaltered in the mutant as well.

**Figure 6:**
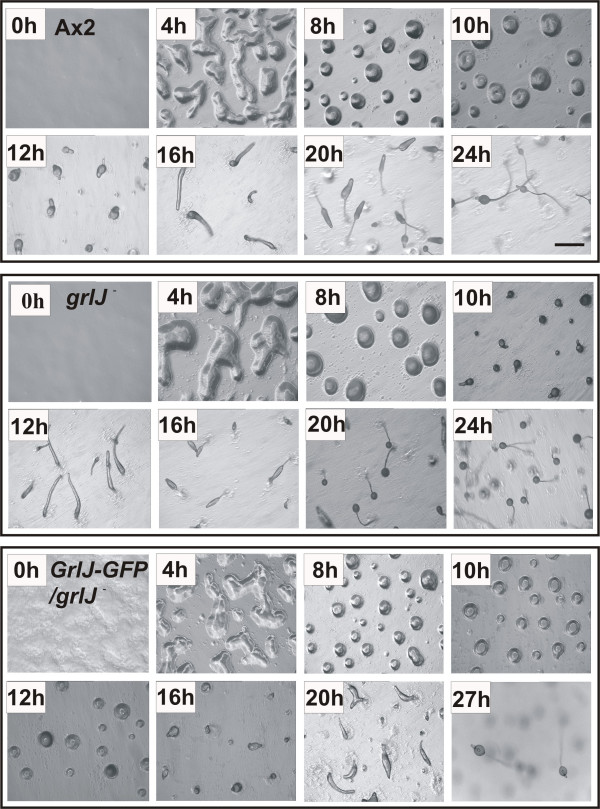
**Development on phosphate agar plates**. Axenically grown cells (A×2, *grlJ*^- ^and *grlJ*^- ^expressing GrlJ-GFP) were plated at a density of 5 × 10^6 ^cells/cm2 and were monitored throughout development. Images were captured every 4 hours using a stereomicroscope. The mutant develops faster after the aggregation stage and forms slugs already after 12 hours of development. Reexpression of GrlJ-GFP in *grlJ*^- ^restored the development. Bar, 1 mm.

**Figure 7:**
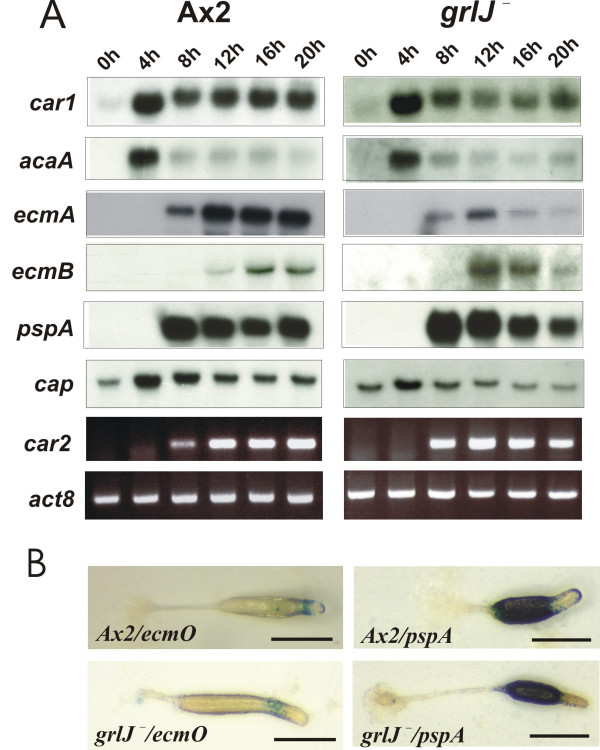
**(A) Prespore and prestalk specific gene expression is altered in *grlJ*^-^**. Total RNA was isolated from A×2 and *grlJ*^- ^at the indicated time points. 20 μg RNA were separated on 1.2 % agarose gels under denaturing conditions (6% formaldehyde) and transferred to membranes as described [54]. cDNA probes specific for the indicated transcripts were used for hybridisation. A CAP cDNA probe was used as a control. The CAP specific message shows an increase during early aggregation and then returns to the level of growing cells [55]. Additionally, RNA from the above time course experiment was used to generate single stranded cDNA (see Methods) and RT-PCR was carried out for analysis of the expression of the stalk specific gene *carB *(cyclic AMP receptor 2). The actin8 gene was amplified as a control. (**B) The *grlJ***^- ^**strain has no pattern formation defect**. *grlJ*^- ^cells were transfected using electroporation with the ecmB-Gal and pspA(D19)-Gal plasmids separately. The transformants were selected with 4 μg/ml G418 and 3 μg/ml of blasticidin. Single colonies were obtained by replica plating and they were developed on a nitrocellulose filter until the slug and culminant stages and then processed for LacZ expression as described [50]. Bar, 200 μm.

As GrlJ is expressed throughout the development with a induction of the expression at later developmental stages we studied whether its loss affects development of the mutant. Starvation was carried out on phosphate agar plates. *grlJ*^- ^cells aggregated well and formed mounds similar to the wild type cells but exhibited an accelerated development after the aggregation stage and slugs were formed at 12 h, culminated at 16 h and formed relatively smaller fruiting bodies within 18 to 20 h (Fig. 6). The expression pattern of cell-type specific developmental markers confirmed these observations. The pattern of transcription for the early aggregation specific genes car1 and acaA was similar in wild type and mutant. There was however an accelerated expression of the prestalk specific gene *ecmB *and another prestalk specific gene, *carB *(*car2*), whereas the levels of *ecmA *and the prespore specific *pspA *transcripts were timely (Fig. 7A). The different regulation of prestalk specific genes may be explained by the findings that they are regulated by different transcription factors [19]. We also tested aggregation in monolayer and observed timely formation of aggregation centres with thick streams for *grlJ*^- ^comparable to the wild type cells (data not shown). Expression of GrlJ-GFP in the mutant led to normal progression during development (Fig. 6).

The chemotactic response to folate in *D. discoideum *requires G-proteins and is expected to be mediated by a yet unidentified GPCR. When we tested GrlJ null cells they responded normally to folate both during growth and development.

### Premature expression of adhesion proteins in *grlJ*^-^

**Figure 8:**
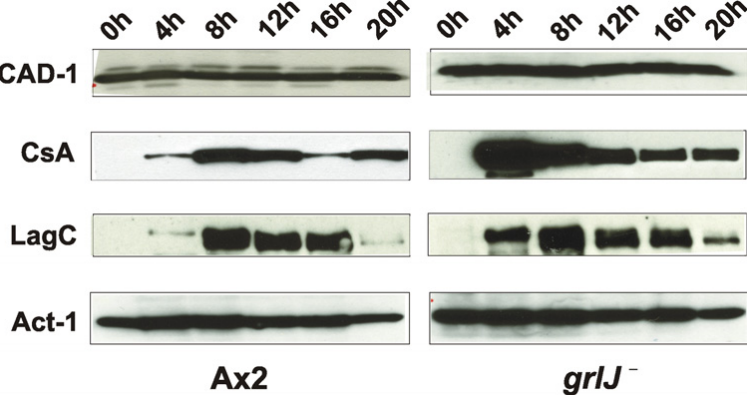
**Expression of cell adhesion molecules in *grlJ*^-^**. Protein samples were obtained at different time points during vegetative growth and development from A×2 cells and *grlJ*^- ^mutants. The equivalent of 2 × 10^5 ^cells was loaded per lane. They were separated by SDS-PAGE and transferred to nitrocellulose using tank blotting. The blots were then probed with polyclonal antibodies specific for DdCAD-1 or gp-150 (LagC) [21,23]. csA was recognized by mAb 33-294-17 [22]. Whereas DdCAD-1 expression was not affected, csA and LagC were expressed earlier. For control the blots were probed for actin.

*Dictyostelium *multicellularity is maintained by expression of several cell adhesion systems. During the initiation of development, DdCAD-1 (gp24), a small, secreted glycoprotein, mediates EDTA-sensitive cell-cell adhesion [20,21]. At the onset of aggregation, expression of contact sites A (csA)/gp80 leads to EDTA-resistant cell-cell adhesion [22,23], and gp150/LagC mediates EDTA-resistant cell-cell adhesion in the post-aggregation stages [24,25]. The temporal regulation of expression was not altered in the *GrlJ*^- ^strain. DdCAD-1 was expressed throughout the developmental timecourse followed by csA at the onset of starvation and LagC. For csA and LagC we noticed however comparatively higher levels at the onset of aggregation (4 h time point) (Fig. 8). These observations may be explained by data from csA mutant analysis that suggested that the expression of one cell adhesion molecule is coupled to the transcriptional control of another [25]. The time point of early aggregation was not noticeably altered in *GrlJ*^-^.

### Post-aggregation events in *grlJ*^-^

During post-aggregative development *D. discoideum *cells undergo characteristic changes and differentiate into different cell types which sort to specific regions in the multicellular organism. We also generated A×2 and *grlJ *^- ^strains carrying plasmids that allowed the expression of the β-galactosidase gene under the control of the *ecmO *and *pspA *promoter (*pspA-Gal*, *ecmO-Gal*) to study pattern formation during development. The EcmO promoter is active in a subpopulation of the prestalk cells, pspA is a well characterised prespore specific marker [26]. We did not note significant differences in the staining pattern of the β-galactosidase driven by either promoter between the mutant and wild-type strains pointing at the defect in the timing of the expression of the developmental markers but not in pattern formation (Fig. 7B).

### *grlJ*^- ^forms longer slugs that break apart during migration

**Figure 9:**
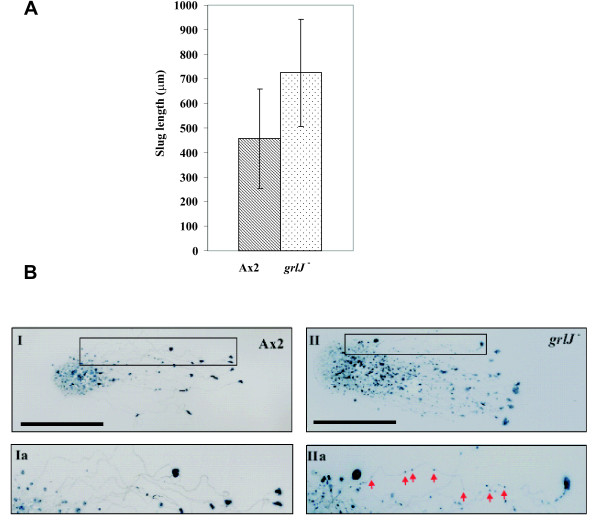
**(A) The *grlJ*^- ^strain shows alterations at the slug stage**. For determination of the slug size, slugs were photographed with an inverted stereomicroscope and around 50 slugs per strain were marked with the DISKUS software program that allowed measuring uneven objects. p value, ≤ 0.005. (**B) Slug migration during phototaxis**. A×2 and *grlJ*^- ^cells were harvested and used for a phototaxis experiment. Both strains formed slugs phototaxing towards the light source. Figure Ia depicts the enlarged version of the box in I, which shows the path of the migrating A×2 slugs. IIa is an enlarged portion of the box in II which displays the pattern of slug movements in *grlJ*^-^. As clearly indicated by the red arrows, the *grlJ*^- ^slug broke several times on its way. Bar, 1 cm.

**Figure 10:**
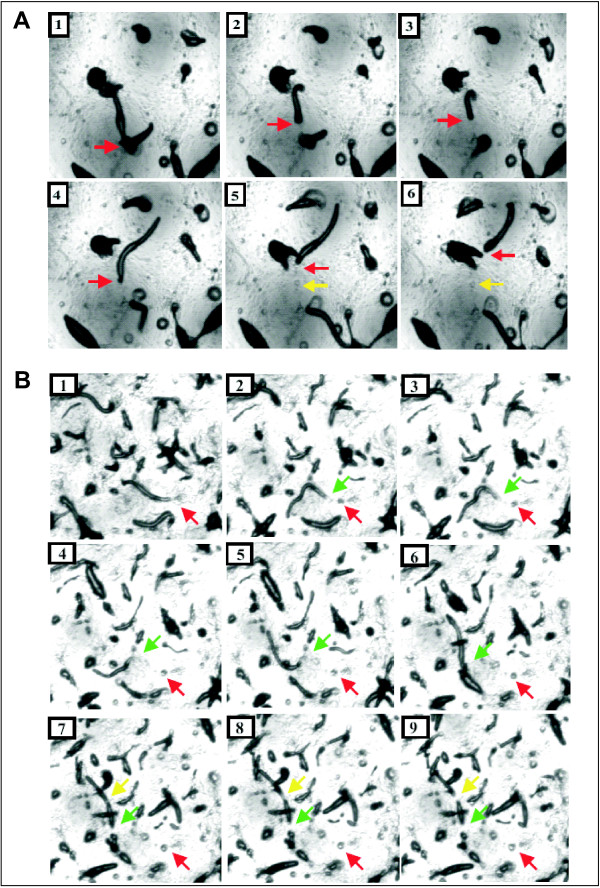
**(A) Movie clips from developing wild type A×2 cells**. The images shown in the figure are still images taken from a series of frames during the slug migration stage. 1, 2, 3, 4, 5 and 6 represent the frames selected that display slug migration. Red arrows indicate the rear portion of the slug under investigation whereas the yellow arrow points to the region after the slug has migrated. The arrows depict the migration of a single representative slug. (**B) Movie clips from developing *grlJ*^- ^slugs**. The images shown are still images taken from a series of frames during the slug migration stage. 1, 2, 3, 4, 5 and 6, 7, 8 and 9 represent the frames selected that display slug migration. Red arrows in all the frames indicate the portion of slug that was left behind whereas green arrows signify the rear end of the slug after it had left behind the first portion and yellow arrows depict the new breaking point. All the arrows show the slug breaking phenomenon of one representative slug.

*Dictyostelium *slugs migrate until they find a favourable condition to culminate. The prestalk cells are comparatively more motile than the prespore cells and they form the tip of the slug acting as a signalling centre and organiser [27]. A very high level of interaction and coordination occurs between the two cell types in the migrating slugs and they have to make a coordinated decision about the right time to stop migration and start culmination [28]. The *grlJ*^- ^strain produced slightly longer slugs (Fig. 9A), which could sense light and phototax. However, they did not migrate as far as wild type slugs and migrated with a wider angle towards the light (59° +/-12) in comparison to the wild type (27° +/-9). Furthermore, we observed the breaking up of slugs in the *grlJ*^- ^strain, which is clearly viewed in the enlarged version indicated by the arrows pointing towards the breaks just in the path of one migrating slug (Fig. 9B). This pattern was observed for most of the slugs traced. We applied live cell microscopy wherein we captured time-lapse images of the *grlJ*^- ^development and compared it with the wild-type development (Fig. 10A,B). The slugs indeed broke several times on their way while migrating.

### Defective sporulation in *grlJ*^-^

**Figure 11:**
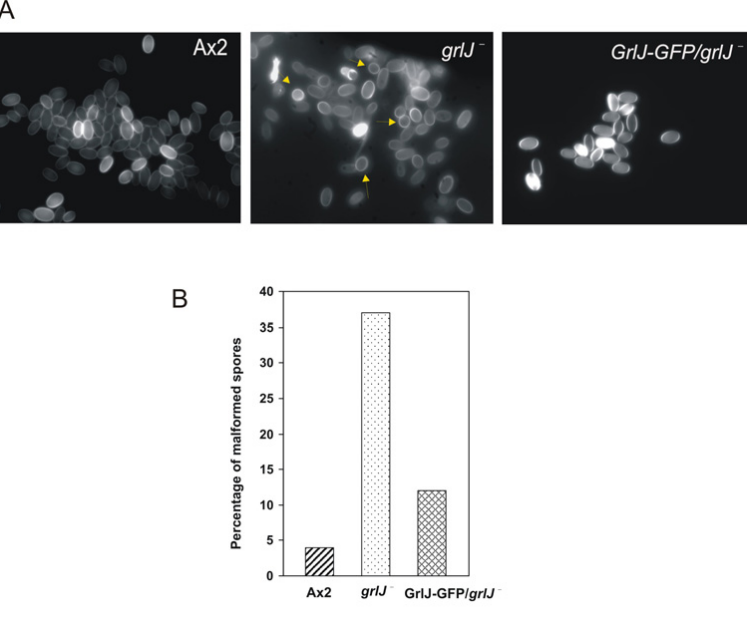
**Spore morphology in *grlJ*^-^**. Spores obtained after 48 h of starvation from the respective strains were stained with 0.1% calcoflour for 10 min and observed under a fluorescent microscope **(A)**. Rounded and misshaped spores were observed for the *grlJ*^- ^strain, whereas in *grlJ*^- ^expressing GrlJ-GFP spores exhibited a normal shape as seen in the parent A×2. The percentage of malformed spores is indicated in the lower panel **(B)**.

Sporulation is a tightly controlled process that involves cell-type differentiation with morphogenesis wherein the prespore cells appear early in development and progressively accumulate components of the spore coat in prespore vesicles which are secreted to assemble the spore coat during encapsulation [29]. Most of the strains that complete development faster produce abnormal spores with reduced viability [30]. Because lack of GrlJ resulted in precocious development following mound formation we studied the spore morphology by staining spores with calcofluor, a fluorescent compound that selectively binds to cellulose present in the spore coat. A×2 cells produced well-elongated slightly banana-shaped spores, whereas 35–40% of *grlJ*^- ^spores were round or misshaped. The abnormality in the spore morphology was complemented by the expression of GrlJ-GFP in the mutant (Fig. 11).

The spore viability in the mutants was analysed and compared with A×2 wild type by plating equal amounts of axenically growing cells on phosphate agar plates and allowing them to develop for 48 hours until spore formation was complete. Spores were harvested and counted to determine the number of spores produced in comparison to the initial number of cells plated. Additionally detergent resistance was checked. The wild type cells showed recovery of 135 % spores from the cells initially plated and they were all viable and resistant to detergent treatment. *grlJ *^- ^also produced spores comparable to the wild type spores (144 %) per cells originally plated. However, fewer spores were viable in *grlJ *^- ^(~30 % reduction) and they were comparatively more sensitive to detergent treatment. This defect in the viability could be rescued by expressing GrlJ-GFP in *grlJ *^- ^cells (Table 4).

**Table 4:** Spore viability assay

**Strains**	**Harvested spores**	**Viable spores**	**Detergent resistant spores**
**A×2**	135% ± 1.03	97% ± 15.7	103% ± 8.33
***grlJ***^-^	144% ± 0.68	63 % ± 6.78	44% ± 7.81
**GrlJ-GFP/*grlJ***^-^	123% ± 0.57	93% ± 11.77	92% ± 11.49

Equal numbers (5 × 10^7^) of cells were plated onto phosphate agar plates and allowed to develop completely until 48 hours. The spores were then harvested and counted. 100 spores per each strain were plated in association with *K. aerogenes *with and without treatment with 0.5% Triton-X-100 for 15 min. The percentage of plaques obtained with/without detergent and the percentage of spores harvested from the mutants was calculated setting the spores harvested from wild type cells as 100 percent. The result shown is from a representative experiment.

Spore formation is controlled by two peptides, **s**pore **d**ifferentiation **f**actor 1 and 2 (SDF-1 and SDF-2). SDF-1 accumulates during the slug stage and is released in a single burst at the onset of culmination, whereas SDF-2 is released at the mid-culmination stage also in a single burst [31]. We assayed for the production of SDF-1 and SDF-2 in *grlJ*^- ^and found that it not only produced comparable levels of both factors but also responded to these factors by inducing sporulation as in wild type (Table 5). SDF-2 is homologous to the human diazepam binding inhibitor (DBI) neuropeptides [32], which can bind to GABA_B _receptors. GABA and glutamate were recently reported to be involved in the regulation of spore formation as inducer and inhibitor, respectively [9]. Both molecules seem to act through GrlE, another member of the family 3 of GPCRs, leading to the induction or inhibition of SDF-2 formation. Some GPCRs of the family 3 form homodimers while others form obligatory heterodimers. The response to GABA and glutamate of GrlJ null cells was therefore tested in a spore formation assay (Table 5). *grlJ *^- ^responded to both GABA and glutamate like the wild type cells ruling out the possibility of being a receptor for either signals and of forming obligatory heterodimers with GrlE.

**Table 5:** Induction of sporulation in *grlJ*^-^

**Strains**	**SDF-1 in sorus**	**SDF-2 in sorus**	**Fold induction of spore formation**			
			
			**SDF-1**	**SDF-2**	**GABA**	**Glutamate**
Wild-type*	10^3 ^U	5–20 10^3 ^U	3 ± 0.7	3.3 ± 0.2	3.3 ± 0.2	1.2 ± 0.3
***grlJ***^-^	10^3 ^U	10^4 ^U	3 ± 0.3	3.2 ± 0.4	3.2 ± 0.2	1.3 ± 0.2

Culminants of each strain were harvested and dissociated in 1 ml cAMP buffer, washed once and counted. 10^5 ^cells were plated in 6 well plates in the presence of the indicated factors. The proportion of spores was determined under the microscope 2 h after induction and quantified as fold induction in comparison to uninduced cells. * Either A×2 or A×4: A×2 was initially used for induction by SDF-1 or SDF-2 [30]. A×4 is used for all factors and responds like A×2 to SDF-1 and SDF-2.

## Discussion

The *Dictyostelium *repertoire of seven transmembrane receptors has been discussed repeatedly [7,33]. Here we have focused specifically on the family 3 and carried out additional analysis using all currently available data which allowed a much more detailed description of the proteins than had been possible before (Table 1). Our further studies were concerned with GrlJ.

Lack of GrlJ alters the developmental pattern from the post-aggregation stages onwards. The *grlJ*^- ^strain aggregates well and forms mounds at a fairly comparable time as the wild type cells. There is a boost in the development thereon and the *grlJ*^- ^strain forms mature fruiting bodies before 18–20 h of starvation. The precocious development was reflected at the protein and mRNA level of post-aggregative genes. Previous studies have found that the expression of post-aggregation genes is directly controlled by the G-Box binding factor GBF [34]. The increase in the expression level of the cell adhesion molecule LagC in the absence of GrlJ suggests that GrlJ normally acts as a negative regulator of development possibly through down regulation of GBF activity. Precocious development has been noted in a variety of *D. discoideum *mutants. Many of them are affected in the cAMP metabolism [35], others like DmtA, DimA and DimB code for components of the DIF signaling pathway [36-38], or are directly involved in spore formation [39].

Several precociously developing strains such as *regA*^- ^or *rdeA*^- ^are sporogenous and end up forming spores with abnormal morphology [40]. The rdeA null strain forms round spores but only when the vegetative cells were grown in the presence of glucose as carbon source [41]. In general, precocious sporulation is mediated by an increase in the intracellular cAMP levels or occurs in strains with an increased PKA activity [30,42]. *grlJ*^- ^produced similar numbers of spores as A×2 wild type, they were however less viable both with and without treatment with detergent pointing to an inefficient maturation. The *grlJ*^- ^cells produced also comparable amounts of the spore differentiation factors SDF-1 and SDF-2 at different culminating stages and were also able to induce sporulation in response to these factors. Likewise, sporulation in *grlJ*^- ^in response to GABA and glutamate was unaltered.

Our current understanding of GrlJ suggests a role in events pertaining to post-aggregation development in *Dictyostelium *as a negative regulator of development. GrlJ is not involved in GABA, glutamate or folate mediated signalling. However, there are many unexplored molecules and secreted factors that are known to act via GPCRs and any amongst them may be a putative ligand for the receptor under investigation or other yet unexplored GPCRs. Such compounds isolated from *Dictyostelium *are for instance the acylated amino sugar derivatives furanodictine A and B possessing neuronal differentiation activity in rat PC-12 cells [43]. Other interesting candidates are the acyl alpha pyronoids having inhibitory effects on the development of *Dictyostelium *[44]. However not much is known about the mechanisms involved therein, but GrlJ can be a putative candidate receptor for any one of these compounds as the loss of GrlJ prevents a regulatory mechanism which is normally occuring and ensuring the correct developmental timing of morphogenetic events. In this respect GrlJ seems to resemble its mammalian counterparts of metabotropic GPCRs which are involved in inhibitory signaling in the brain.

## Conclusion

GrlJ is a seven transmembrane protein belonging to the family3 of GPCRs, that contains the GABA_B_/glutamate like receptor family. In the brain GABA (gamma-amino-butyric acid) is the principal inhibitory neurotransmitter and signals through ionotropic and metabotropic receptor systems At its N-terminus it carries a BMP-domain which is characteristic for most of the members of this family and is also found in the mammalian homologs. GrlJ transcripts are present throughout *Dictyostelium *development, GFP-tagged GrlJ localises to the plasma membranes and to internal membranes in the perinuclear area which partially overlap with ER membranes. GrlJ deficient cells showed a precocious development indicative of an inhibitory role of GrlJ during this stage of development. Alterations were also observed during later development and resulted in defective slug formation and altered spore viabilty. We propose that GrlJ is an important regulator of *Dictyostelium *development.

## Methods

### *Dictyostelium *cell culture, vector construction and mutant isolation

*Dictyostelium *wild type and mutant cells were cultured as described in [14]. For generation of the gene disruption vector, the Neomycin resistance cassette was retrieved as an EcoRV fragment from pDNeo2 [45] and inserted into the EcoRV site of a genomic clone encoding the GrlJ gene. Screening of the transformants was carried out using a PCR approach [46], confirmation was done by Southern blot analysis. Rescue experiments were carried out with a vector encoding blasticidin resistance which allowed expression of full length GrlJ carrying GFP at its C-terminus under control of the actin15 promoter (generously provided by R. Blau-Wasser).

### RNA isolation and quantitation, northern blotting

Total RNA was extracted from A×2 and *grlJ*^- ^at different developmental stages or from different assay conditions using the Qiagen RNeasy Mini kit. The manufacturer's protocol for the isolation of RNA from the cytoplasm of animal cells was used for preparation. The RNA samples were used directly for northern blot analysis or after reverse transcription for RT-PCR (Real-Time PCR). cDNA was generated using the M-MLV reverse transcriptase, RNAse H minus (Roche) according to the manufacturers protocol. Usually 1–5 μg of the respective total RNA was used for each RT reaction. cDNAs generated were used as a template to carry out PCR with the respective gene specific primers. Primers were chosen using a freely available progam [46]. For quantitative Real-Time PCR primers were selected such that the expected product size was between 250–500 bp. Prior to use in Real-Time PCR experiments the quality of the cDNA and the primers were tested by PCR. Real-Time PCR was carried out with the QuantiTect SYBR green PCR kit (Qiagen) according to the manufacturer's protocol. For each sample gene specific primers (10 pmole) and 1 μl of cDNA were used. As a quantification standard defined concentrations (10 ng, 1 ng, 100 pg, 10 pg and 1 pg) of GrlJ's C-terminal gene sequences in pGEMTeasy were used. The experiments were carried out using an Opticon Real-Time PCR machine. Calculations were done using the delta-delta CT method. Actin specific primers were used as positive control and to ensure comparable concentrations of cDNA in samples of wild type and mutant cells. For comparative analysis of the transcript levels of all Grls, a semiquantitative Real-Time PCR analysis was carried out. Equal amounts of cDNA were used for all experiments and PCR was carried out for 25 cycles. This allowed a comparison of the relative transcript levels of all Grls. Northern blot analysis was carried out using total RNA isolated at different time points [47].

### Development on phosphate-agar or water agar plates

Cells at a density of 2–3 × 10^6 ^cells/ml were washed twice with Soerensen phosphate buffer, pH 6.0 [47]. 5 × 10^7 ^cells were then resuspended in 1 ml Soerensen phosphate buffer and evenly distributed onto a phosphate-buffered agar plate (9 cm in diameter) and incubated at 21°C. Different stages of development were observed and the images were captured using a stereomicroscope at the indicated time points.

### Phototaxis [48]

Axenically growing cells were harvested by centrifugation at 2000 rpm for 2 minutes and washed twice in water before placing 10^6 ^cells each on 1% water agar plates. The plates were incubated in a petridish storage containers (thermocool box with walls painted completely black from within) containing a vertical 3 mm wide perforation along the length of the container in constant subdued light for 48 hours at 22°C. The slime trails left behind the migrating slugs were blotted onto nitrocellulose membranes and stained with 0.1% amido black in 25% isopropanol and 10% acetic acid (staining solution) for 10 minutes, destained in 25% isopropanol and 10% acetic acid and washed with water and air dried.

### Spore viability assay [[49], modified]

Equal numbers (5 × 10^7^) of cells (A×2, *grlJ*^-^) were plated onto phosphate agar plates and spores formed at 48 h were harvested and counted, respectively. They were then treated with 0.5% Triton-X-100 for 15 min and diluted with Soerensen buffer and 100 spores each were plated onto SM agar plates in association with *K. areogenes*. Detergent resistant spores were counted as the number of plaques formed once they appeared and calculated for the viability. The experiments were carried out in triplicates.

### Spore induction assay and quantification of produced SDFs factors [31]

Spore induction is performed with cells taken from developing structures. 10^7 ^cells of each strain were washed and allowed to develop on filters till they reached early to mid-culminant stage (approximately 20 h for most strains, 16 h for *grlJ*^-^) then harvested in 1 ml cAMP buffer (without cAMP) (10 mM MES, pH 6.5, 10 mM NaCl, 10 mM KCl, 1 mM CaCl_2_. 1 mM MgSO_4_), centrifuged and washed twice in 1 ml cAMP buffer. 10^5 ^cells each were plated in 6 wells plates with 2 ml cAMP buffer plus or minus various factors: 10 nM synthetic SDF-1, 10 pM synthetic SDF-2, 100 nM GABA, 10 nM glutamate. The proportion of spores was determined under the microscope after 1 h of incubation for SDF-2 or GABA and 2 h for SDF-1. The fold induction corresponds to the ratio between the level of spores observed plus and minus factors. The amount of SDF-1 and SDF-2 accumulated in terminal structures was determined as previously described [31,32]. Briefly, fruiting bodies corresponding to 10^7 ^cells are dissociated in 1 ml cAMP buffer. Cells are removed by two centrifugations at 6000 rpm. Aliquots of the supernatants were incubated with either cation (C-50) or anion (A-25) exchange resins to trap SDF-1 and SDF-2 respectively. SDF-1 and SDF-2 activity are then determined by serial dilution on test KP cells (strain constitutive for PKA-C) in a sporogenous assay. One unit corresponds to the lowest dilution giving full induction of spore formation. The number of units are standardized as per 10^3 ^producing cells.

### LacZ reporter gene expression

A×2 cells and *grlJ*^- ^cells were transformed using electroporation with the ecmO-Gal and pspA (D19)-Gal plasmids separately. The transformants were selected with 4 μg/ml G418 for wild type cells whereas additional 3 μg/ml of blasticidin was added for *grlJ*^- ^cells. Single colonies were obtained by replica plating and they were developed on a nitrocellulose filter until slug and culminant stages and then processed for LacZ expression as described [50].

### Miscellaneous methods

Immunofluorescence microscopy and cell fractionation studies were done as described in [14]. For western blot analysis of protein samples from developing cells samples were prepared at the respective time points of *Dictyostelium *development and separated by SDS-PAGE gels (8%, 10% or 15% respectively) were blotted on to nitrocellulose membranes. They were blocked with 5 % milk in 1× NCP and probed with different dilutions of the respective primary antibodies and POD-conjugated secondary antibodies. They were then detected by ECL (enhanced chemiluminescence) reactions.

## Authors' contributions

Y.P. carried out this work and wrote the manuscript. A.N. initiated the project and oversaw it at all stages, R. M. was involved in the intial steps of this project and carried out the transcriptional analysis of the family3 proteins in *D. discoideum*. C.A. carried out part of the spore analysis. All authors have read and approved the final manuscript.
